# The relationship between radiation-induced G_1_arrest and chromosome aberrations in Li-Fraumeni fibroblasts with or without germline *TP53* mutations

**DOI:** 10.1054/bjoc.2001.1896

**Published:** 2001-07

**Authors:** J M Boyle, A Spreadborough, M J Greaves, J M Birch, J M Varley, D Scott

**Affiliations:** CRC Department of Cancer Genetics, Paterson Institute for Cancer Research, CRC Christie Research Centre, Manchester, M20 4BX; CRC Paediatric and Familial Cancer Research Group, Royal Manchester Children's Hospital, Manchester, M27 1HA, UK

**Keywords:** Li-Fraumeni syndrome, p53, Chk2, ionizing radiation, G_1_arrest, chromosome aberrations, fibroblasts

## Abstract

We previously showed that cultured fibroblasts from patients with the cancer-prone Li-Fraumeni (LF) syndrome, having heterozygous germline *TP53* mutations, sustain less ionizing radiation-induced permanent G_1_ arrest than normal fibroblasts. In contrast, fibroblast strains from LF patients without *TP53* mutations showed normal G_1_ arrest. We have now investigated the relationship between the extent of G_1_ arrest and the level of structural chromosome damage (mainly dicentrics, rings and acentric fragments) in cells at their first mitosis after G_1_ irradiation, in 9 LF strains with *TP53* mutations, 6 without *TP53* mutations and 7 normal strains. Average levels of damage in the mutant strains were 50% higher than in normals, whereas in non-mutant LF strains they were 100% higher. DNA double strand breaks (dsb) are known to act as a signal for p53-dependent G_1_ arrest and to be the lesions from which chromosome aberrations arise. These results suggest that a minimal level of dsb is required before the signal for arrest is activated and that p53-defective cells have a higher signal threshold than p53-proficient cells. Dsb that do not cause G_1_ blockage can progress to mitosis and appear as simple deletions or interact to form exchange aberrations. The elevated levels in the non-mutant strains may arise from defects in the extent or accuracy of dsb repair. In LF cells with or without *TP53* mutations, the reduced capacity to eliminate or repair chromosomal damage of the type induced by ionising radiation, may contribute to cancer predisposition in this syndrome. © 2001 Cancer Research Campaign http://www.bjcancer.com


*British Journal of Cancer* (2001) **85**(2), 293–296
© 2001 Cancer Research Campaign
doi: 10.1054/ bjoc.2001.1896, available online at http://www.idealibrary.com on IDEAL®

### The relationship between radiation-induced $G_1$ arrest and chromosome aberrations in Li-Fraumeni fibroblasts with or without germline *TP53* mutations

**JM Boyle[1], A Spreadborough[1], MJ Greaves[1], JM Birch[2], JM Varley[1] and D Scott[1]**

[1]CRC Department of Cancer Genetics, Paterson Institute for Cancer Research, CRC Christie Research Centre, Manchester M20 4BX; [2]CRC Paediatric and Familial Cancer Research Group, Royal Manchester Children's Hospital, Manchester M27 1HA, UK

**Summary** We previously showed that cultured fibroblasts from patients with the cancer-prone Li-Fraumeni (LF) syndrome, having heterozygous germline *TP53* mutations, sustain less ionizing radiation-induced permanent $G_1$ arrest than normal fibroblasts. In contrast, fibroblast strains from LF patients without *TP53* mutations showed normal $G_1$ arrest. We have now investigated the relationship between the extent of $G_1$ arrest and the level of structural chromosome damage (mainly dicentrics, rings and acentric fragments) in cells at their first mitosis after $G_1$ irradiation, in 9 LF strains with *TP53* mutations, 6 without *TP53* mutations and 7 normal strains. Average levels of damage in the mutant strains were 50% higher than in normals, whereas in non-mutant LF strains they were 100% higher. DNA double strand breaks (dsb) are known to act as a signal for p53-dependent $G_1$ arrest and to be the lesions from which chromosome aberrations arise. These results suggest that a minimal level of dsb is required before the signal for arrest is activated and that p53-defective cells have a higher signal threshold than p53-proficient cells. Dsb that do not cause $G_1$ blockage can progress to mitosis and appear as simple deletions or interact to form exchange aberrations. The elevated levels in the non-mutant strains may arise from defects in the extent or accuracy of dsb repair. In LF cells with or without *TP53* mutations, the reduced capacity to eliminate or repair chromosomal damage of the type induced by ionising radiation, may contribute to cancer predisposition in this syndrome. © 2001 Cancer Research Campaign http://www.bjcancer.com

**Keywords**: Li-Fraumeni syndrome; p53; Chk2; ionizing radiation; $G_1$ arrest; chromosome aberrations; fibroblasts

When normal human fibroblasts are exposed in vitro to ionizing radiation, a proportion of the cells in the $G_1$ phase of the cell cycle suffer a dose-dependent reduction in their ability to progress further through the cell cycle (Little and Nagasawa, 1985). We have shown (Williams et al, 1997; Boyle et al, 1999) that the extent of this permanent $G_1$ arrest is less pronounced in fibroblasts of patients with the Li-Fraumeni (LF) syndrome carrying inherited, heterozygous mutations of the *TP53* gene which confer a high risk of cancer (reviewed by Varley et al, 1997). Our results are consistent with the hypothesis that p53 is responsible for monitoring the integrity of the genome (Lane, 1992) and eliminating genetically damaged cells that might otherwise be predisposed to malignant conversion.

In some cell types, apoptosis appears to be the functional equivalent of $G_1$ arrest and is reduced or eliminated in p53-defective cells after exposure to DNA-damaging agents (references in Williams et al, 1996, 1997; Camplejohn et al, 1995). For example, a cell line of human lymphocyte origin carrying a *TP53* mutation showed less radiation-induced apoptosis than an isogenic cell line without the mutation (Schwartz et al, 1995) although neither line sustained $G_1$ arrest (Little et al, 1995). The mutant cell line exhibited more structural chromosome damage at the first mitosis after $G_1$ irradiation than the wild type cells and Schwartz and colleagues (Schwartz et al, 1995; Schwartz and Jordan, 1997) suggested that the cells with the most chromosome damage were selectively eliminated by pre-mitotic apoptosis in the p53-proficient line.

The hypothesis of Schwartz et al, if extended to cells with reduced permanent $G_1$ arrest, would predict that fibroblasts of LF patients with germline *TP53* mutations would exhibit more chromosome damage at their first mitosis after $G_1$ irradiation than normal fibroblasts. Only about half of the families with the LF syndrome are found to carry inherited *TP53* mutations (Varley et al, 1997) and we have shown that fibroblasts from non-mutation cases have no defect in $G_1$ arrest after irradiation (Boyle et al, 1999) and might not, therefore, be expected to show enhanced chromosomal radiosensitivity of $G_1$ cells. In the present study we have investigated the $G_1$ chromosomal radiosensitivity of fibroblasts from 7 normal donors, 9 LF patients with *TP53* mutations and 6 without mutations.

### MATERIALS AND METHODS

Full details of the cell strains used are given in Boyle et al (1998, 1999, 2000). The maximum number of in vitro population doublings (PD) was previously established for each strain and all were irradiated before they had reached 80% of their maximum PD, because LF strains tend to become chromosomally unstable during the last 20% of their proliferative lifetime (Boyle et al 1998, 2000).

Designations of the strains, which were all established in this laboratory, are as follows.

Group 1 (normal controls); 83MA, 84MA, 105MA, 120MA, 162MA, 177MA, 187MA.

*Received 25 January 2001*
*Revised 24 April 2001*
*Accepted 26 April 2001*

*Correspondence to:* D Scott

Group 2 (mutation carriers); FH1, 109MA, 110MA, 124MA, 138MA, 178MA, 185MA, 191MA, 193MA. These 9 strains were established from 7 LF families each with different *TP53* mutations (Boyle et al, 1999). LF families have been subdivided into classical (LFS) and LF-like (LFL) depending upon the number of individuals with cancer, the nature of the cancers, their time of onset and other criteria (Varley et al, 1997). Group 2 comprised 5 LFS and 2 LFL families.

Group 3 (non-mutation carriers); 80MA, 107MA, 121MA, 126MA, 128MA, 147MA. The 6 strains were from different LF families (3 LFS and 3 LFL) and all were from patients with experience of cancers typical of those in LF families (e.g. sarcomas and breast cancer), except strain 121MA which was from an unaffected brother of a patient with a typical LF cancer. Strain 121MA was included because at late passage (>90% maximum PD) it showed the spontaneous chromosomal instability seen in ageing LF strains from cases with typical LF-type cancers (Boyle et al, 2000).

To produce a $G_1$ population, cells were grown to confluence in Minimal Essential Medium with Earle's salts (MEM) and 15% fetal calf serum (FCS) without antibiotics and maintained at confluence with twice weekly medium changes for 10–14 days. 2 days before irradiation the medium was changed to MEM with 0.1% FCS. On the day of irradiation, cells were harvested by trypsinisation, counted, and exposed in suspension (approximately $1 \times 10^6$ per ml) to 0 or 3 Gy $^{137}$Cs gamma irradiation at a dose rate of 3.3 Gy per min. Cells were then seeded at $1 \times 10^6$ per T150 flask (Corning) in complete medium. At 16 h after seeding, before cells had entered their first mitosis (Williams et al, 1997), colcemid (150 ng ml$^{-1}$) was added to arrest cells at their first metaphase and incubation was continued for a further 50 h at which time metaphase preparations were made by normal procedures. Cells were stained with Giemsa.

For each cell strain 50–100 metaphases were scored for structural aberrations from coded slides of irradiated and non-irradiated cells with 45 or 46 chromosomes.

Measurement of permanent $G_1$ arrest was made previously (Williams et al, 1997; Boyle et al, 1999). Briefly, $G_1$ cells were irradiated or mock-irradiated, subcultured in medium containing tritiated thymidine to label cells entering the S phase, harvested at intervals between 60–120 hours post-irradiation and prepared for autoradiography. The extent of permanent arrest was determined by comparing the proportions of labelled cells in irradiated and non-irradiated samples.

The Mann–Whitney U test was used to test the significance of differences in endpoints between groups of strains.

### RESULTS

Mean aberration yields and the range of values for irradiated and non-irradiated cells of each of the 3 groups are given in Table 1, together with values for the degree of permanent $G_1$ arrest.

The spontaneous aberration yield in the normal stains was approximately 5% and was slightly, but not significantly, higher in the other 2 groups. Further details of spontaneous structural and numerical aberrations in these strains as they progress to senescence are given in Boyle et al (1998, 2000).

The majority of induced aberrations (subtracting the spontaneous yields) were of the chromosome type, comprising dicentrics, centric rings and excess acentric fragments (see footnote 4 of Table 1), typical of cells irradiated in $G_1$. Approximately 15% of the induced aberrations were of the chromatid type (mainly breaks and large gaps), possibly indicating the presence of a small proportion of cells in S and $G_2$ at the time of irradition and/or arising from DNA base damage in $G_1$ cells (Schwartz and Russell, 1999).

As we reported earlier (Williams et al, 1997; Boyle et al, 1999), the average degree of $G_1$ arrest was significantly less ($P < 0.01$) in mutation-carrying strains (group 2, 44.6 ± 16.3%) than in normals (group 1, 75.0 ± 13.0%). In contrast, the mean frequency of total aberrations induced in group 2 strains (74.4 ± 12.7 per 100 cells) was approximately 50% *higher* ($P$ = 0.02) than in normals (50.0 ± 16.2). This difference was seen for all types of aberrations.

The average extent of $G_1$ arrest in non-*TP53* mutation-carrying strains (group 3, 73.0 ± 7.4%) was very similar ($P$ = 0.67) to that of normals (75.0 ± 13.0%), as previously reported (Boyle et al, 1999). However, the mean yield of total aberrations (98.7 ± 42.6 per 100 cells) in group 3 was almost 100% higher ($P < 0.01$) than in normals (50.0 ± 16.2), for all aberration types (Table 1).

**Table 1** Chromosomal aberration yields and the extent of G1 arrest in the 3 groups of cell strains

| Group | Dose (Gy) | Chromosome-type aberrations[1] | | | | Chromatid aberrations | Total aberrations | G1 arrest (%)[2] |
|---|---|---|---|---|---|---|---|---|
| | | Dicentrics | Rings | Acentrics[4] | Total | | | |
| 1. Normals (*n* = 7) | 0 | 0 ± 0 | 0 ± 0 | 1.3 ± 1.3 (0 – 3) | 1.3 ± 1.3 (0 – 3) | 3.2 ± 2.1 (0 – 6) | 4.6 ± 2.2 (2 – 8) | – |
| | 3 | 17.1 ± 15.5 (4 – 44) | 5.4 ± 5.4 (0 – 16) | 19.6 ± 8.1 (12 – 34) | 42.1 ± 19.5 (20 – 68) | 11.1 ± 5.8 (5 – 18) | 53.1 ± 20.4 (30 – 85) | 75.0 ± 13.0 (50.2 – 90.9) |
| 2. Mutation carriers (*n* = 9)[5] | 0 | 0.67 ± 1.4 (0 – 4) | 0 ± 0 | 1.0 ± 2.6 (0 – 8) | 1.7 ± 2.7 (0 – 8) | 3.6 ± 2.9 (0 – 10) | 5.2 ± 3.8 (2 – 14) | |
| | 3 | 29.4 ± 15.0 (14 – 59) | 7.9 ± 2.5 (4 – 12) | 29.0 ± 15.9 (4 – 54) | 66.3 ± 12.4 (54 – 88) | 15.0 ± 9.7 (6 – 26) | 81.0 ± 14.7 (64 – 114) | 44.6 ± 16.3 [3] (14.9 – 63.5) |
| 3. Non-mutation carriers | 0 | 0 ± 0 | 0 ± 0 | 1.3 ± 1.6 (0 – 4) | 1.3 ± 1.6 (0 – 4) | 6.3 ± 4.1 (2 – 14) | 7.7 ± 4.1 (6 – 16) | – |
| (*n* = 6) | 3 | 39.7 ± 20.9 (16 – 72) | 10.7 ± 6.2 (4 – 20) | 37.0 ± 10.3 (22 – 50) | 87.3 ± 27.7 (58 – 140) | 19.7 ± 17.8 (6 – 58) | 106.3 ± 46.5 (66 – 198) | 73.0 ± 7.4 (58.7 – 79.4) |

[1] Aberration yields are expressed per 100 cells ± SD. Ranges are given in brackets. 50–100 cells scored from each sample except for irradiated cells from normal strain 187MA where only 30 cells could be analysed. [2] Data from Boyle et al (1999). [3] G1 arrest data for 185MA are not included because they were obtained after a dose of 4.0 Gy (Varley et al, 1998), but the 3.0 Gy data for 178MA are from the same family. [4] One acentric fragment was allocated to each dicentric and ring. The remaining (excess) acentric fragments are given in this column. [5] Details of mutations given in Boyle et al (1999) except for strain 185MA, which was from family 2635 (see Table 1 in Boyle et al, 1999).

 © 2001 Cancer Research Campaign

Because of the wide range of aberration yields in group 3, giving a comparatively large standard deviation, the mean yield was not significantly greater than that of group 2 ($P = 0.15$). Within group 3, strain 80MA showed a considerably higher frequency of both spontaneous (16/100 cells) and induced (182/100 cells) aberrations than the 5 remaining strains, all of which had a spontaneous yield of 6/100 cells and a range of induced yields from 60–96/100 cells. The mean frequency in the 5 strains was 82.0 ± 13.4 per 100 cells which was still significantly higher ($P = 0.01$) than the normals. Strain 80MA also had the lowest level of $G_1$ arrest (58.7%) amongst group 3, the remaining 5 of which had a range from 72.8 to 79.4% (mean 73.0 ± 7.4%). Strain 80MA has now been found to carry a germline mutation in the *hCHK2* gene (Bell et al, 1999) which is likely to be involved in $G_1$ checkpoint control because of its influence on p53 transcription (see Discussion).

### DISCUSSION

Our results support the hypothesis of Schwartz and colleagues (Schwartz et al, 1995; Schwartz and Jordon, 1997) that p53-proficient cells have a mechanism for selectively eliminating cells with high levels of chromosome damage induced in the $G_1$ phase of the cell cycle. However, whereas in the cells of lymphoid origin used by Schwartz et al this selection process occurs by apoptosis, in human fibroblasts the mechanism appears to involve permanent $G_1$ arrest. During the course of our studies, Schwartz and Russell (1999) have reported finding increased levels of chromosome damage at the first mitosis after gamma-irradiation of a human adenocarcinoma cell line in which permanent $G_1$ arrest was eliminated by transfection with the human papillomavirus type 16 E6 gene which inactivates p53 expression.

There is abundant evidence that radiation-induced DNA double strand breaks (dsb) can act as a signal for p53-dependent permanent $G_1$ arrest (references in Schwartz and Russell, 1999). Our observations and those of Schwartz and Russell on non-lymphoid cells provide additional support for this concept, since radiation-induced chromosome aberrations arise mainly from dsb (reviewed by Pfeiffer et al, 2000). These results suggest that a certain minimal level of dsb is required before the signal for arrest is activated and that p53-defective cells have a higher signal threshold than p53-proficient cells. Dsb that do not cause $G_1$ blockage can progress to mitosis and appear as simple deletions (excess fragments) or interact to for, exchange aberrations (e.g. dicentrics and rings). Schwartz and Russell (1999) argue that the increase in chromatid aberrations, which they also observed in irradiated HPV16-E6 cells compared with non-transfected cells, is a consequence of deficient base excision repair that has been reported in p53-defective human fibroblasts (Ford et al, 1998).

There have been several other studies (Parshad et al, 1993, Lee et al, 1994, Williams, et al, 1997), including our own (Wang et al, 1996, Mitchell and Scott, 1997, Varley et al, 1998) in which p53-defective cells have been found to have elevated levels of radiation-induced structural chromosome damage compared with p53-proficient cells. However, in these investigations, cells were either irradiated as asynchronous populations or in the $G_2$ phase of the cell cycle. In the former, the specific role of $G_1$ arrest in determining these increased yields could not be assessed. The latter results demonstrate that in p53-defective cells it is not only those that are in the $G_1$ phase at the time of irradiation that are at increased risk of enhanced chromosome damage, but the mechanism of $G_2$ radiosensitization is at present unknown.

There is compelling evidence that the degree or accuracy of DNA double strand break repair can determine the levels of chromosome damage in cells exposed to ionizing radiation (Pfeiffer et al, 2000). We suggest that defects in these mechanisms may explain the elevated levels of chromosome damage in cells strains without *TP53* mutations and with normal $G_1$ arrest. However, for strain 80MA, with a mutation in the *hCHK2* gene, impaired $G_1$ arrest is a more likely explanation since it had the lowest $G_1$ arrest value of the group 3 strains, overlapping the group 2 range. The Chk2 protein is activated in response to ionizing radiation (Tominaga et al, 1999) and is required to stabilize p53 transcription (Chehab et al, 2000) so will have an influence on $G_1$ arrest. The fact that the induced aberration frequency in 80MA is almost twice as high as that of the most sensitive *TP53* mutation-carrying strain suggests that Chk2 has a function in addition to the stabilization of p53 and that this additional function influences the aberration production after irradiation. However, this interpretation of the abnormal behaviour of strain 80MA must remain tentative until the other non-*TP53* mutant strains have been tested for germline *hCHK2* mutations. So far, only strain 107MA has been tested and found to be without a mutation of this gene.

In Li-Fraumeni cells with or without *TP53* mutations, a reduced capacity to eliminate or repair chromosomal damage of the type induced by ionizing radiation, from endogenous or exogenous sources, may contribute to cancer predisposition in this syndrome.

### ACKNOWLEDGEMENT

This work was funded by the Cancer Research Campaign.

### REFERENCES

Bell DW, Varley JM, Szydlo T, Kang DH, Wahrer DCR, Shannon KE, Lubrotavitch M, Versilis SJ, Isselbacher KI, Birch JM, Li FP, Garber JE and Haber DA (1999) Heterozygous germline mutations in hCHK2 are a cause of Li-Fraumeni syndrome. *Science* **286**: 2528–2531

Boyle JM, Mitchell ELD, Greaves MJ, Roberts SA, Tricker K, Varley JM, Birch JM and Scott D (1998) Chromosome instability is a predominant trait of fibroblasts from Li-Fraumeni families. *Br J Cancer* **77**: 2181–2192

Boyle JM, Greaves MJ, Camplejohn RS, Birch JM, Roberts SA, and Varley JM (1999) Radiation-induced $G_1$ arrest is not defective in fibroblasts from Li-Fraumeni families without *TP53* mutations. *Br J Cancer* **79**: 1657–1664

Boyle JM, Spreadborough A Greaves MJ, Birch JM and Scott D (2000) Chromosome instability in fibroblasts derived from Li-Fraumeni syndrome families without TP53 mutations, *Br J Cancer* **83**: 1136–1138

Camplejohn RS, Perry P, Hodgson SV, Turner G, Williams A, Upton C, MacGeoch C, Mohammed S and Barnes DM (1995) A possible screening test for inherited p53-related defects based on apoptotic response of peripheral blood lymphocytes to DNA damage. *Br J Cancer* **72**: 654–662

Chehab NH, Malikzay A, Appel M and Halizonetis TD (2000) Chk2/hCds1 functions as a DNA damage checkpoint in $G_1$ by stabilizing p53. *Genes Dev* **14**: 278–288

Ford JM, Baron EL and Hanawalt PC (1998) Human fibroblasts expressing the human papillomvirus *E6* gene are deficient in global genomic nucleotide excision repair and sensitive to ultraviolet irradiation. *Cancer Res* **58**: 599–603

Lane DP (1992) P53, guardian of the genome. *Nature* **358**: 15–16

Lee JM, Abrahamson JLA, Kandel R, Donehower LA and Bernstein A (1994) Susceptibility to radiation-carcinogenesis and accumulation of chromosomal breakage in p53 deficient mice. *Oncogene* **9**: 3731–3736

Little JB and Nagasawa H (1985) The effect of confluent holding on potentially lethal damage repair, cell cycle progression, and chromosomal aberrations in human normal and ataxia-telangiectasia fibroblasts. *Radiation Res* **101**: 81–93

Little JB, Nagasawa H, Keng PC, Yu Y and Li C-Y (1995) Absence of radiation-induced G1 arrest in two closely related human lymphoblast cell lines that differ in p53 status. *J Biol Chem* **270**: 11033–11036

© 2001 Cancer Research Campaign 

Mitchell ELD and Scott D (1997) G2 chromosomal radiosensitivity in fibroblasts of ataxia-telangiectasia heterozygotes and a Li-Fraumeni Syndrome patient with radioresistant cells. *Int J Radiat Biol* **72**: 435–438

Parshad R, Price FM, Pirollo KF, Chang EH and Sanford KK (1993) Cytogenetic response to $G_2$-phase X-irradiation in relation to DNA repair and radiosensitivity in a cancer-prone family with Li-Fraumeni syndrome. *Radiation Res* **136**: 236–240

Pfeiffer P, Goedecke W and Obe G (2000) Mechanisms of DNA double-strand break repair and their potential to induce chromosomal aberrations. *Mutagenesis* **15**: 289–302

Schwartz JL and Jordon R (1997) Selective elimination of human lymphoid cells with unstable chromosome aberrations by p53-dependent apoptosis. *Carcinogenesis* **18**: 201–205

Schwartz JL and Russell (1999) The effect of functional inactivation by HPV-E6 transformation on the induction of chromosome aberrations by gamma rays in human tumor cells. *Radiat Res* **74**: 747–754

Schwartz JL, Jordon R, Sedita BA, Swenningson MJ, Banath JP and Olive PL (1995) Different sensitivity to cell killing and chromosome mutation induction by gamma rays in two human lymphoblastoid cell lines derived from a single donor: possible role of apoptosis. *Mutagenesis* **10**: 227–233

Tominga K, Morisaki H, Kaneko Y, Fujimoto A, Tanaka T, Ohtsubo M, Hirai M, Okayama H, Ikeda K and Nakanishi M (1999) Role of human Cds1 (Chk2) kinase in DNA damage checkpoint and its regulation by p53. *J Biol Chem* **274**: 31463–31467

Varley JM, Evans DGR and Birch JM (1997) Li-Fraumeni syndrome – a molecular and clinical review. *Br J Cancer* **76**: 2437–2442

Varley JM, Chapman P, McGown G, Thorncroft M, White GRM, Greaves MJ, Scott D, Spreadborough A, Tricker KJ, Birch JM, Evans DGR, Reddel R, Camplejohn RS, Burn J and Boyle JM (1998) Genetic and functional studies of a germline TP53 splicing mutation in a Li-Fraumeni family, *Oncogene* **16**: 3291–3298

Wang L, Cui Y, Lord BI, Roberts SA, Potten CS, Hendry JH and Scott D (1996) Gamma-ray-induced cell killing and chromosome abnormalities in the bone marrow of p53-deficient mice. *Radiation Res* **146**: 259–266

Williams AC, Miller JC, Collard T, Browne SJ, Newbold RF and Paraskeva C (1997) The effect of different TP53 mutations on the chromosomal stability of a human colonic adenoma cell line with endogenous wild type TP53 activity, before and after DNA damage. *Genes Chromosomes Cancer* **20**: 44–52

Williams KJ, Heighway J, Birch JM, Norton JD and Scott D (1996) No defect in $G_1$/S cell cycle arrest in Li-Fraumeni lymphoblastoid cell lines. *Br J Cancer* **74**: 698–703

Williams KJ, Boyle JM, Birch JM, Norton JD and Scott D (1997) Cell cycle arrest defect in Li-Fraumeni syndrome: a mechanism of cancer predisposition? *Oncogene* **14**: 277–282

*© 2001 Cancer Research Campaign*
